# Influence of Preoperative Serum Aspartate Aminotransferase (AST) Level on the Prognosis of Patients with Non-Small Cell Lung Cancer

**DOI:** 10.3390/ijms17091474

**Published:** 2016-09-03

**Authors:** Shu-Lin Chen, Ning Xue, Mian-Tao Wu, Hao Chen, Xia He, Jian-Pei Li, Wan-Li Liu, Shu-Qin Dai

**Affiliations:** State Key Laboratory of Oncology in South China, Collaborative Innovation Center for Cancer Medicine, Sun Yat-sen University Cancer Center, Guangzhou 510060, China; chenshl@sysucc.org.cn (S.-L.C.); xuening@sysucc.org.cn (N.X.); wumiant@sysucc.org.cn (M.-T.W.); chenhao@sysucc.org.cn (H.C.); hexia@sysucc.org.cn (X.H.); lijianpeisysucc@sina.com (J.-P.L.)

**Keywords:** aspartate aminotransferase (AST), recurrence-free survival (RFS), overall survival (OS), prognosis, non-small cell lung cancer (NSCLC)

## Abstract

The purpose of this work is to analyze preoperative serum aspartate aminotransferase (AST) levels and their effect on the prognosis of patients with non-small cell lung cancer (NSCLC) after surgical operation. These analyses were performed retrospectively in patients with NSCLC followed by surgery; participants were recruited between January 2004 and January 2008. All clinical information and laboratory results were collected from medical records. We explored the association between preoperative serum AST and recurrence-free survival (RFS), and the overall survival (OS) of NSCLC patients. Kaplan–Meier analysis and Cox multivariate analysis, stratified by the AST median value, were used to evaluate the prognostic effect. A chi-squared test was performed to compare clinical characteristics in different subgroups. A *p*-value of ≤0.05 was considered to be statistically significant. A total of 231 patients were enrolled. The median RFS and OS were 22 and 59 months, respectively. The AST levels were divided into two groups, using a cut-off value of 19 U/L: High AST (>19 U/L), *n* = 113 vs. low AST (≤19 U/L), *n* = 118. Multivariate analysis indicated that preoperative serum AST > 19 U/L (hazard ratio (HR) = 0.685, 95% confidence interval (CI): 0.493–0.994, *p* = 0.046 for RFS, HR = 0.646, 95% CI: 0.438–0.954, *p* = 0.028 for OS) was an independent prognostic factor for both RFS and OS. High preoperative serum AST levels may serve as a valuable marker to predict the prognosis of NSCLC after operation.

## 1. Introduction

Lung cancer is one of the most malignant tumors and has the highest morbidity and mortality rates of any cancer worldwide. According to statistics, the United States in 2013 alone had 228,190 new cases of lung cancer, and the number of related deaths was 159,480, thus ranking first in terms of death [1]. The occurrence of lung cancer in China is more prevalent. According to recent data from the National Cancer Prevention and Control Research Office, the incidence of lung cancer in China over the past 18 years has increased year-on-year, with an average annual growth rate of 1.63% [2].

According to pathological type, lung cancer can be divided into non-small cell lung cancers (NSCLC) and small cell lung cancers (SCLC), of which NSCLC accounts for 80%–85% of the cases [3]. With the development of surgical techniques and molecularly-targeted drugs, the overall efficacy of treatment of NSCLC has greatly improved; however, there are large differences in the prognoses of patients with different genetic variations and physiological conditions. Finding a method to evaluate the prognosis of NSCLC patients is important for clinical treatment. The serum levels of alanine aminotransferase (ALT) and aspartate aminotransferase (AST) are distributed in the liver [4]. After trauma, ischemia, hypoxia, cell membrane integrity, and function damage can be seen an increase in cell permeability, mitochondrial swelling, and rupture of the cells (causing ALT and AST release into the blood stream). In the case of hemodynamic instability, the aforementioned manifestations will become more serious. There is a correlation between an increase in liver serum aminotransferase and liver damage in patients with closed abdominal injury, and there is also a close relationship between the level of serum aminotransferase and the severity of liver injury. ALT and AST are produced by both malignant and non-malignant cells. Compared to normal cells, most cancer cells produce ATP by glycolysis under aerobic conditions rather than via the tricarboxylic acid cycle. Glycolysis is necessary in cancer cells to produce the ATP and anabolic precursors required for survival, growth, and invasion. AST functions in tandem with malate dehydrogenase to transfer electrons from nicotinamide adenine dinucleotide (NADH) across the inner mitochondrial membrane, which is closely related to glycolysis. So, AST may be associated with cancer prognosis. Additionally, some studies have confirmed that ALT and AST are significantly associated with the prognoses of several cancers, such as hepatocellular carcinoma, renal cell carcinoma, colonic, pancreatic, and breast cancer [5,6,7,8,9]. However, the prognostic value of the preoperative serum AST in patients with NSCLC is not clear. In this study, our aim is to evaluate if the level of serum AST at the preoperative phase in patients with NSCLC can serve as a prognostic marker for outcome.

## 2. Results

### 2.1. Patient Characteristics

The pretreatment characteristics of 231 NSCLC patients are listed in Table 1. There were 160 men (69.26%) and 71 women (30.74%), with a median age of 55 years (range, 30–80 years). The most common histology was adenocarcinoma (118, 51.08%), followed by squamous cell carcinoma (SCC) (88, 38.10%), and other histologies (25, 10.82%). Of all patients, 99 (42.86%) were stage I, 47 (20.35%) were stage II, 77 (33.33%) were stage III, and 8 (3.46%) were stage IV. At the time of the last follow-up, 119 patients (51.5%) had developed recurrences after treatment.

### 2.2. Relation of Patient Recurrence-Free Survival (RFS) and Overall Survival (OS) to AST Levels

**Figure 1:**
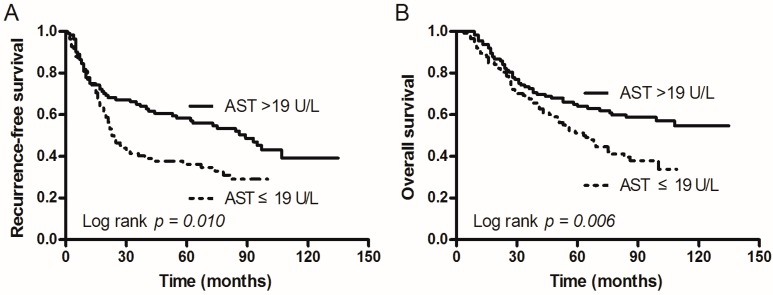
Kaplan–Meier survival curves of non-small cell lung cancer (NSCLC) patients were divided into two groups (AST > 19 U/L and AST ≤ 19 U/L). (**A**) Recurrence-free survival (RFS) of patients with AST. The survival of patients with AST ≤ 19 U/L was shorter than that of patients with AST > 19 U/L (*p* = 0.010); (**B**) Overall survival (OS) of patients with AST. The survival of patients with AST ≤ 19 U/L was also shorter than that of patients with AST > 19 U/L (*p* = 0.006). AST: aspartate aminotransferase.

The 231 included patients were categorized into the following two groups according to their AST levels: The first group (AST levels ≤19 U/L) and the second group (AST levels >19 U/L). The mean RFS of the first group was 45.437 months (95% confidence interval (CI): 37.405–53.469 months) vs. the second group, 76.137 months (95% CI: 65.207–87.067 months; Table 2). The mean OS of the first group was 64.624 months (95% CI: 57.282–71.967 months; Table 2) vs. the second group, 91.878 months (95% CI: 82.276–101.480 months). Kaplan–Meier estimates of RFS and OS for patients with different AST levels are shown in Figure 1. The results show that patients with elevated AST levels were significantly associated with longer RFS and OS (log-rank test: *p* = 0.010 and *p* = 0.006, respectively), and patients with low AST levels had a significantly poorer prognosis.

### 2.3. Univariate and Multivariate Analyses of Prognostic Factors

Univariate and multivariate analyses of RFS (Table 3) and OS (Table 4), using 19 U/L as a cut-off value, hazard ratios (HRs), and 95% CI estimated from Cox regression models indicated that the AST (HR 0.646; 95% CI 0.438–0.954; *p* = 0.028) was strongly associated with OS and RFS (HR 0.685; 95% CI 0.473–0.994; *p* = 0.046). In addition to elevated AST levels, platelet-to-lymphocyte ratio (PLR) was significantly associated with RFS (HR 1.714; 95% CI 1.187–2.476; *p* = 0.004). Multivariate analyses indicated that elevated AST levels were a highly significant predictor for RFS and OS. It thus serves as an independent prognostic factor in NSCLC patients.

### 2.4. The Distribution of Clinical Characteristics in the AST Subgroup

Further analyses were conducted by comparing the distribution of clinical characteristics in the AST subgroup, shown in Table 5. We found that tumor size (*p* = 0.032), ALT (*p* = 0.000), and PLR (*p* = 0.018) were significantly different between the two groups. However, the level of AST was not significantly correlated with other clinical characteristics.

### 2.5. Subgroup Analysis According to Tumor Size, ALT, and PLR

**Figure 2:**
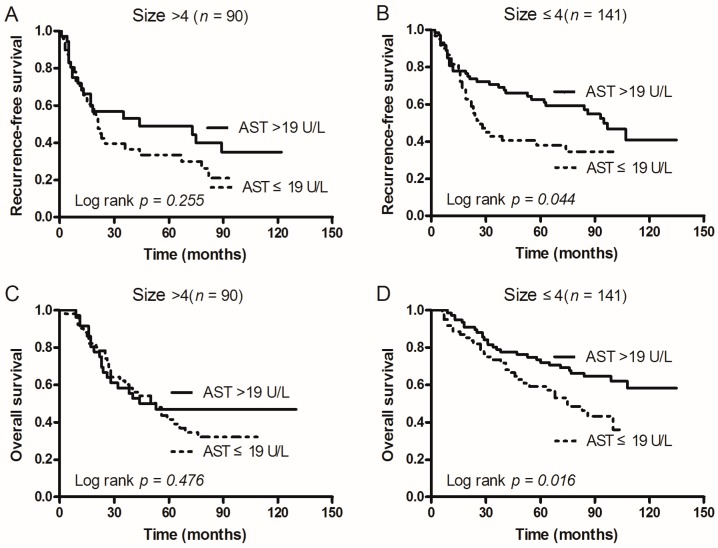
Kaplan–Meier survival curves of patients with AST > 19 U/L and AST ≤ 19 U/L grouped by patient tumor size. (**A**) In tumors size >4 cm patients, RFS of patients with AST ≤ 19 U/L was shorter than that of those with AST > 19 U/L (*p* = 0.255); (**B**) In size ≤4 cm patients, RFS of patients with AST ≤ 19 U/L was shorter than that of those with AST > 19 U/L (*p* = 0.044); (**C**) In size >4 cm patients, OS of patients with AST ≤ 19 U/L was shorter than that of those with AST > 19 U/L (*p* = 0.476); (**D**) In size ≤4 cm patients, OS of patients with AST ≤ 19 U/L was shorter than that of those with AST > 19 U/L (*p* = 0.016).

**Figure 3:**
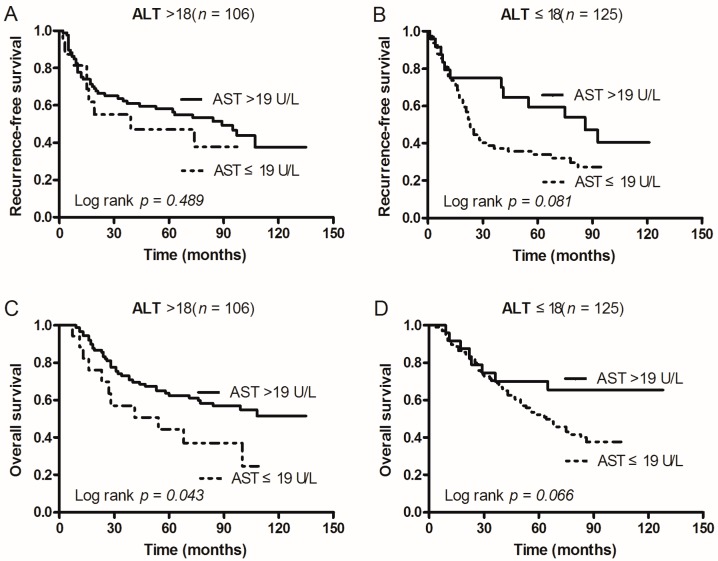
Kaplan–Meier survival curves of patients with AST > 19 U/L and AST ≤ 19 U/L grouped by patient subgroups of ALT. (**A**) In ALT > 18 U/L patients, RFS of patients with AST ≤ 19 U/L was shorter than that of those with AST > 19 U/L (*p* = 0.489); (**B**) In ALT ≤ 18 U/L patients, RFS of patients with AST ≤ 19 U/L was shorter than that of those with AST > 19 U/L (*p* = 0.081); (**C**) In ALT > 18 U/L patients, OS of patients with AST ≤ 19 U/L was shorter than that of those with AST > 19 U/L (*p* = 0.043); (**D**) In ALT ≤ 18 U/L patients, OS of patients with AST ≤ 19 U/L was shorter than that of those with AST > 19 U/L (*p* = 0.066).

**Figure 4:**
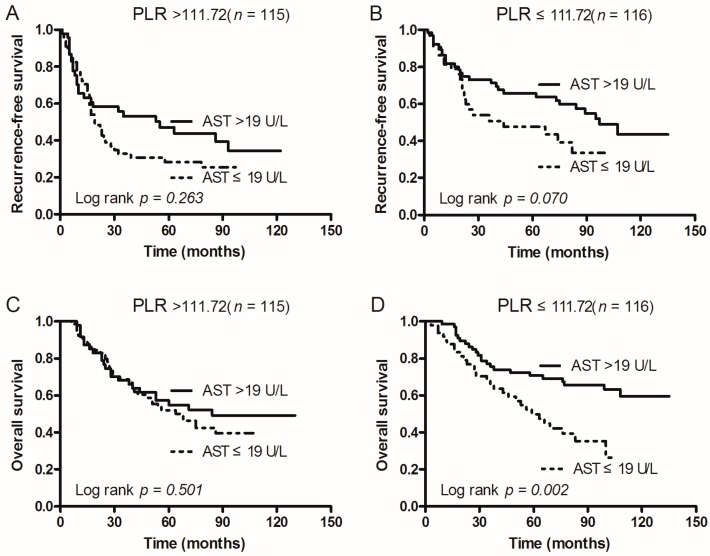
Kaplan–Meier survival curves of patients with AST > 19 U/L and AST ≤ 19 U/L grouped by patient subgroups of PLR. (**A**) In PLR > 111.72 patients, RFS of patients with AST ≤ 19 U/L was shorter than that of those with AST > 19 U/L (*p* = 0.263); (**B**) In PLR ≤ 111.72 patients, RFS of patients with AST ≤ 19 U/L was shorter than that of those with AST > 19 U/L (*p* = 0.070); (**C**) In PLR > 111.72 patients, OS of patients with AST ≤ 19 U/L was shorter than that of those with AST > 19 U/L (*p* = 0.501); (**D**) In PLR ≤ 111.72 patients, OS of patients with AST ≤ 19 U/L was shorter than that of those with AST > 19 U/L (*p* = 0.002).

In order to investigate the subgroups of patients that were positively influenced by preoperative AST, we divided patients according to tumor size, ALT, and PLR. In patients with AST > 19 U/L: Both RFS and OS after operation were significantly better when tumor size was ≤4 cm (*p* = 0.044 and 0.016), but not for patients with a tumor size of >4 cm (*p* = 0.255 and *p* = 0.476; Figure 2). In addition, OS was only better when patients had ALT > 18 U/L (*p* = 0.043; Figure 3C) and PLR ≤ 111.72 (*p* = 0.002; Figure 4D). However, RFS was not significantly influenced by ALT levels (*p* = 0.489 vs. 0.081; Figure 3) and PLR values (*p* = 0.263 vs. 0.070; Figure 4).

## 3. Discussion

In the present study, we performed a retrospective evaluation of the prognostic significance of AST in NSCLC patients. We found that elevated preoperative serum AST levels were associated with a good clinical outcome in NSCLC patients, and Cox regression analysis showed that AST was an independent prognostic factor of both poor RFS and overall survival OS. Blood-based measurement of AST and ALT was described as a useful tool to evaluate hepatocellular carcinoma prognosis [10]. Shen et al. also reported that preoperative AST-to-platelet ratio was an independent prognostic factor for hepatitis B-induced hepatocellular carcinoma, after hepatic resection [5]. Susan et al. analyzed 312 patients with liver metastases from breast cancer and found that AST was the single most important prognostic factor for survival after the diagnosis of liver metastases [11]. Additionally, a report modeling prognostic factors in advanced pancreatic cancer found that the AST level was associated with pancreatic cancer prognosis [8]. Recently, some researchers have applied AST/ALT as a significant prognostic factor in patients with non-metastatic renal cell carcinoma [6,12]. Our previous studies have shown that ALT/AST ratio was associated with prognosis in patients undergoing curative treatment for gastric cancer [13]. In this study, we analyzed the correlation between clinical characteristics and AST subgroups. The results showed that tumor size, ALT, and PLR were significantly correlated with AST. Then, we investigated whether the subgroups of patients were positively influenced by preoperative AST. We found that for patients with AST > 19 U/L, both RFS and OS were significantly better when tumor sizes were ≤4 cm; there was no difference in patients with tumor sizes >4 cm. In addition, OS was better only when patients had ALT > 18 U/L and PLR ≤ 111.72. However, RFS was not significantly influenced by ALT and PLR. Previous studies have reported that PLR and NLR (neutrophil-to-lymphocyte ratio) serve as independent predictors of survival in patients with NSCLC [14,15]. In contrast, we did not observe that PLR and NLR were predictors of OS, while PLR emerged as an independent predictor associated with RFS, and there was no significant trend for NLR to be a risk factor for tumor relapse. A possible explanation for this is as follows: first, we used median values of PLR and NLR as cut-off points to divide the high and low groups, which is different from other reports; second, only Chinese NSCLC patients were included in our study, whereas other reports were mostly focused on Caucasian populations. Whether there are differences between Asian and Caucasian populations—which are required for further validation in a larger population from different races—is unknown. Third, the values of platelets, neutrophils, and lymphocytes were tested using different detection systems, which could affect the results. Serum AST and ALT activities were used as markers of liver damage [16]. In human tissues, both cytoplasmic AST and mitochondrial AST proteins consist of a compartment-specific homodimer that is encoded by two separate genes, *GOT1* and *GOT2*, respectively [17]. According to the current view, the impact of AST on cancer relapse and survival rate is unclear. Researcher insight into the potential role of AST in human carcinogenesis remains speculative. In contrast to normal cells, most cancer cells rely on aerobic glycolysis to generate the energy needed for cellular processes. Warburg assumed that there was mitochondrial dysfunction in cancer cells, as he observed that cancer cells could convert most glucose into lactate, regardless of the availability of oxygen [18]. Some studies showed that cancer cell proliferation could also obtain energy through glutamine metabolism, which is necessary for tumor cells to maintain nucleotide biosynthesis and non-essential amino acids, which are catalyzed by AST and ALT [19,20,21,22]. Thornburg et al. reported that oxamate could inhibit the proliferation of transformed breast adenocarcinoma cells in vitro, and AST acts as an essential metabolic target of oxamate [9]. Although there were important discoveries revealed by this study, there were also some limitations. First, it should be noted that this study has only examined a small sample size and is a single-center study—research should be done on a large scale and using multicenter prospective validation of NSCLC groups; second, we only focused on the impact of preoperative serum albumin (ALB), ALT, AST, NLR, and PLR on the prognosis of patients with NSCLC, but other traditional prognostic factors, such as cytokeratin 19 fragment (CYFRA21-1) [23], carcinoembryonic antigen (CEA) [24], SCC antigen [24], and cancer antigen 125 (CA 125) [25], were not available in this study. Thus, we were unable to determine whether preoperative AST was a better predictor of RFS and OS compared with traditional tumor markers; third, we found that AST level was an independent prognostic factor in NSCLC, based on a retrospective review. The mechanism of how AST affects the survival time of patients should be further explored in depth. Despite these limitations, we report that the level of preoperative serum AST was correlated with RFS and OS in patients with NSCLC. In conclusion, this analysis was performed retrospectively in patients with NSCLC. The prognostic value of AST was evaluated in patients with NSCLC, and the relationships between serum AST and clinical pathological parameters were analyzed. The results showed that a high preoperative serum AST level is an independent risk factor affecting RFS time and OS of patients, and AST was associated with tumor sizes, ALT, and PLR. The results show that the pre-operative levels of serum AST in patients with NSCLC can be used to determine prognosis. Additional studies are required for further validation in a larger population from a multi-center study.

## 4. Materials and Methods

### 4.1. Patients

We performed a retrospective review of patients undergoing surgical treatment of NSCLC between January 2004 and January 2008. A total of 231 patients with NSCLC were enrolled in this study. Patient clinical data, including age, sex, smoking, family history, BMI, diagnosis, Pathological Tumor Node Metastasis stage according to the 6th Edition of the TNM Classification at the time of pre-operative phase, ALB, ALT, AST, NLR, PLR and follow-up results were collected and recorded. The last follow-up was on 30 June 2015. This study was approved by the Sun Yat-sen University Cancer Center research ethics committee (Identification code: GZR2014-106; Date 28 February 2014). All patients provided written informed consent for their medical information to be stored and used in the hospital database at their first visit to our center.

### 4.2. Inclusion Criteria

(1) Patients were diagnosed as having primary bronchial lung cancer by means of surgical biopsy. The pathological type was NSCLC (adenocarcinoma, SCC, other NSCLC); (2) Clinical data were completed; (3) Before the physical examination, routine blood treatment of patients after admission and a biochemical examination were performed.

### 4.3. Exclusion Criteria

(1) Lung cancer which was not been pathologically diagnosed; (2) Patients with other malignant tumors; (3) Patients with severe organ system diseases, such as severe infections, cardiovascular diseases, hepatitis, etc.; (4) Patients with incomplete clinical data.

### 4.4. Clinical Outcomes Assessment and Patient Follow-up

The primary endpoint of our study was RFS. The secondary endpoint was OS. We calculated RFS from the date of surgery to the date of the first relapse at any site, death from any cause or the date of the last follow-up visit. OS was calculated from the date of the surgery to the date of death from any cause or patient censoring at the date of the last follow-up.

### 4.5. Statistical Analysis Method

All clinical and experimental data were entered into a computer by hand. Statistical analysis was done using SPSS 19 (IBM, Chicago, IL, USA) for Windows. The cutoff values of preoperative ALT, AST, PLR, and NLR were estimated by the median, and the cutoff value of ALB was 35 g/L. For group comparisons, chi-square and Fisher’s exact tests were used for categorical variables. Survival curves were constructed according to the Kaplan–Meier method and compared using the log-rank test. Multivariate Cox regression was used to perform the survival analysis in order to estimate the HR for various factors. Results of the Cox regression analyses were reported with HR, together with the corresponding 95% CI. The difference was statistically significant when *p* < 0.05.

## Figures and Tables

**Table 1:** General characteristics of the patient population (*n* = 231).

Characteristics	*n* (%)
Age (years)	
>55	115 (49.78)
≤55	116 (50.22)
Sex	
Male	160 (69.26)
Female	71 (30.74)
Smoking behavior	
Yes	132 (57.14)
No	99 (42.86)
Family history of cancer	
Yes	34 (14.72)
No	197 (85.28)
BMI (kg/m^2^)	
≥25	42 (18.18)
<25	189 (81.82)
Histology type	
AC	118 (51.08)
SCC	88 (38.10)
OTH	25 (10.82)
pTNM stage	
I	99 (42.86)
II	47 (20.35)
III	77 (33.33)
IV	8 (3.46)
Maximum tumor diameter (cm)	
>4	90 (38.96)
≤4	141 (61.04)
Lymph node status	
NEG	128 (55.41)
POS	103 (44.59)
ALB (g/L)	
>35	225 (97.40)
≤35	6 (2.60)
ALT (U/L)	
>18	106 (45.89)
≤18	125 (54.11)
AST (U/L)	
>19	113 (48.92)
≤19	118 (51.08)
PLR	
>111.72	115 (49.78)
≤111.72	116 (50.22)
NLR	
>2.14	114 (49.35)
≤2.14	117 (50.65)

BMI: body mass index; pTNM: Pathological Tumor Node Metastasis stage according to the 6th Edition of the TNM Classification; AC: adenocarcinoma; SCC: squamous cell carcinoma; OTH: others; NEG: negative; POS: positive; ALB: albumin; ALT: alanine transaminase; AST: aspartate aminotransferase; PLR: platelet-to-lymphocyte ratio; NLR: neutrophil-to-lymphocyte ratio.

**Table 2:** Clinical and laboratory characteristics of 231 patients associated with overall survival (OS) and recurrence-free survival (RFS).

Patient Characteristics	OS (Months) Mean (95% CI)	*p*	RFS (Months) Mean (95% CI)	*p*
Age (years)				
>55	76.665 (67.565–85.766)	0.359	63.009 (53.369–72.650)	0.329
≤55	86.066 (76.251–95.882)		63.447 (52.182–74.712)	
Sex				
Male	80.981 (72.625–89.337)	0.367	66.107 (56.491–75.724)	0.973
Female	85.290 (73.692–96.888)		62.291 (49.820–74.762)	
Smoking behavior				
Yes	76.098 (67.424–84.772)	0.154	64.175 (54.373–73.978)	0.493
No	87.945 (77.661–98.228)		62.324 (50.469–74.179)	
Family history of cancer				
Yes	90.878 (73.913–107.842)	0.370	73.886 (53.745–94.027)	0.390
No	79.310 (72.120–86.500)		60.293 (52.583–68.004)	
BMI (kg/m^2^)				
≥25	80.675 (64.345–97.006)	0.820	76.185 (56.974–95.397)	0.222
<25	81.451 (74.199–88.703)		58.999 (51.380–66.618)	
Histology type				
AC	85.729 (76.289–95.168)	0.405	63.068 (52.399–73.738)	0.896
SCC	77.553 (67.136–87.971)		63.293 (51.408–75.178)	
OTH	66.185 (48.611–83.760)		66.965 (44.968–88.962)	
pTNM stage				
I	102.736 (93.568–111.905)	0.000	84.840 (73.536–96.144)	0.000
II	67.075 (53.697–80.453)		54.221 (37.712–70.729)	
III	63.093 (51.851–74.335)		45.489 (33.275–57.704)	
IV	47.425 (10.633–26.583)		14.625 (3.929–25.321)	
Maximum tumor diameter (cm)				
>4	68.968 (58.271–79.664)	0.004	51.870 (40.398–63.342)	0.017
≤4	90.734 (82.198–99.270)		72.029 (61.938–82.119)	
Lymph node status				
NEG	97.137 (88.423–105.850)	0.000	80.866 (70.495–91.237)	0.000
POS	62.962 (53.589–72.336)		42.230 (32.059–52.402)	
ALB (g/L)				
>35	83.133 (76.131–90.136)	0.732	65.655 (57.586–73.723)	0.844
≤35	57.167 (32.407–81.926)		55.000 (19.572–90.428)	
ALT (U/L)				
>18	86.252 (76.245–96.258)	0.332	73.243 (61.645–84.840)	0.081
≤18	77.381 (68.506–86.256)		54.699 (45.117–64.282)	
AST (U/L)				
>19	91.878 (82.276–101.480)	0.006	76.137 (65.207–87.067)	0.010
≤19	64.624 (57.282–71.967)		45.437 (37.405–53.469)	
PLR				
>117.2	77.121 (67.702–86.540)	0.236	51.428 (41.478–61.378)	0.003
≤117.2	86.983 (77.387–96.580)		76.815 (65.777–87.853)	
NLR				
>2.14	75.314 (66.031–84.598)	0.168	55.143 (45.256–65.030)	0.076
≤2.14	87.570 (78.010–97.129)		73.198 (61.769–84.626)	

CI: confidence interval.

**Table 3:** Univariate and multivariate analyses of RFS.

Characteristics	Univariate Analysis			Multivariate Analysis		
	HR	95% CI	*p*	HR	95% CI	*p*
Age (years)						
>55/≤55	0.837	0.583–1.201	0.333	-	-	-
Sex						
male/female	1.007	0.683–1.484	0.974	-	-	-
Smoking behavior						
yes/no	0.882	0.615–1.266	0.497	-	-	-
Family history of cancer						
yes/no	0.800	0.478–1.338	0.395	-	-	-
BMI (kg/m^2^)						
≥25/<25	0.734	0.444–1.212	0.227	-	-	-
Histology type						
AC/SCC/OTH	1.001	0.754–1.330	0.993	-	-	-
pTNM stage						
I/II/III/IV	1.626	1.346–1.964	0.000	1.351	1.006–1.814	0.450
Maximum tumor diameter (cm)						
>4/≤4	1.549	1.075–2.232	0.019	1.358	0.939–1.965	0.104
Lymph node status						
NEG/POS	2.406	1.670–3.467	0.000	1.610	0.923–2.807	0.093
ALB (g/L)						
>35/≤35	1.149	0.284–4.654	0.846	-	-	-
ALT (U/L)						
>18/≤18	0.726	0.504–1.045	0.085	-	-	-
AST (U/L)						
>19/≤19	0.623	0.432–0.899	0.012	0.685	0.473–0.994	0.046
PLR						
>111.72/≤111.72	1.728	1.200–2.487	0.003	1.714	1.187–2.476	0.004
NLR						
>2.14/≤2.14	1.383	0.963–1.986	0.079	-	-	-

HR: hazard ratio.

**Table 4:** Univariate and multivariate analyses of OS.

Characteristics	Univariate Analysis			Multivariate Analysis		
	HR	95% CI	*p*	HR	95% CI	*p*
Age (years)						
>55/≤55	1.189	0820–1.724	0.362	-	-	-
Sex						
male/female	1.206	0.800–1.818	0.370	-	-	-
Smoking behavior						
yes/no	1.267	0.867–1.852	0.221	-	-	-
Family history of cancer						
yes/no	0.781	0.453–1.346	0.373	-	-	-
BMI (kg/m^2^)						
≥25/<25	1.055	0.661–1.684	0.821	-	-	-
Histology type						
AC/SCC/OTH	1.106	0.828–1.476	0.495	-	-	-
pTNM stage						
I/II/III/IV	1.572	1.297–1.905	0.000	1.219	0.897–1.656	0.207
Maximum tumor diameter (cm)						
>4/≤4	1.711	1.179–2.484	0.005	1.451	0.987–2.131	0.058
Lymph node status						
NEG/POS	2.373	1.627–3.461	0.000	1.741	0.968–3.130	0.064
ALB (g/L)						
>35/≤35	0.819	0.260–2.584	0.733	-	-	-
ALT (U/L)						
>18/≤18	0.832	0.573–1.209	0.335	-	-	-
AST (U/L)						
>19/≤19	0.594	0.407–0.869	0.007	0.646	0.438–0.954	0.028
PLR						
>111.72/≤111.72	1.250	0.862–1.813	0.239	-	-	-
NLR						
>2.14/≤2.14	1.297	0.894–1.881	0.171	-	-	-

**Table 5:** Comparison of clinical characteristics of patients with different AST (χ^2^ test).

Characteristics	Subcategories	AST	AST	*p*
		>19	≤19	
Age (years)	>55	58	57	0.694
	≤55	55	61	
Sex	male	82	78	0.320
	female	31	40	
Smoking behavior	yes	68	64	0.425
	no	45	54	
Family history of cancer	yes	15	19	0.581
	no	98	99	
BMI (kg/m^2^)	≥25	26	16	0.087
	<25	87	102	
Histology type	AC	60	58	0.829
	SCC	41	47	
	OTH	12	13	
pTNM stage	I–II	75	71	0.343
	III–IV	38	47	
Maximum tumor diameter (cm)	>4	36	54	0.032
	≤4	77	64	
Lymph node status	NEG	65	63	0.597
	POS	48	55	
ALB (g/L)	>35	108	117	0.113
	≤35	5	1	
ALT (U/L)	>18	89	17	0.000
	≤18	24	101	
PLR	>111.72	47	68	0.018
	≤111.72	66	50	
NLR	>2.14	50	64	0.148
	≤2.14	63	54	
